# Chronic schistosomiasis suppresses HIV-specific responses to DNA-MVA and MVA-gp140 Env vaccine regimens despite antihelminthic treatment and increases helminth-associated pathology in a mouse model

**DOI:** 10.1371/journal.ppat.1007182

**Published:** 2018-07-26

**Authors:** Godfrey A. Dzhivhuho, Samantha A. Rehrl, Hlumani Ndlovu, William G. C. Horsnell, Frank Brombacher, Anna-Lise Williamson, Gerald K. Chege

**Affiliations:** 1 Department of Pathology, Faculty of Health Sciences, University of Cape Town, Cape Town, Western Cape, South Africa; 2 Department of Integrative Biomedical Sciences, Faculty of Health Sciences, University of Cape Town, Cape Town, Western Cape, South Africa; 3 Institute of Infectious Disease and Molecular Medicine, Faculty of Health Sciences, University of Cape Town, Cape Town, Western Cape, South Africa; 4 Institute of Microbiology and Infection, University of Birmingham, Birmingham, United Kingdom; 5 Laboratory of Molecular and Experimental Immunology and Neurogenetics, Centre Nationnal de la Recherche Scientifique, University of Orleans and Le Studium Institute for Advanced Studies, Rue Dupanloup, Orléans, France; 6 International Centre for Genomic Engineering and Biotechnology–Cape Town Component, Cape Town, Western Cape, South Africa; 7 South African Medical Research Council, Cape Town, Western Cape, South Africa; Emory University, UNITED STATES

## Abstract

Future HIV vaccines are expected to induce effective Th1 cell-mediated and Env-specific antibody responses that are necessary to offer protective immunity to HIV infection. However, HIV infections are highly prevalent in helminth endemic areas. Helminth infections induce polarised Th2 responses that may impair HIV vaccine-generated Th1 responses. In this study, we tested if *Schistosoma mansoni* (Sm) infection altered immune responses to SAAVI candidate HIV vaccines (DNA and MVA) and an HIV-1 gp140 Env protein vaccine (gp140) and whether parasite elimination by chemotherapy or the presence of Sm eggs (SmE) in the absence of active infection influenced the immunogenicity of these vaccines. In addition, we evaluated helminth-associated pathology in DNA and MVA vaccination groups. Mice were chronically infected with Sm and vaccinated with DNA+MVA in a prime+boost combination or MVA+gp140 in concurrent combination regimens. Some Sm-infected mice were treated with praziquantel (PZQ) prior to vaccinations. Other mice were inoculated with SmE before receiving vaccinations. Unvaccinated mice without Sm infection or SmE inoculation served as controls. HIV responses were evaluated in the blood and spleen while Sm-associated pathology was evaluated in the livers. Sm-infected mice had significantly lower magnitudes of HIV-specific cellular responses after vaccination with DNA+MVA or MVA+gp140 compared to uninfected control mice. Similarly, gp140 Env-specific antibody responses were significantly lower in vaccinated Sm-infected mice compared to controls. Treatment with PZQ partially restored cellular but not humoral immune responses in vaccinated Sm-infected mice. Gp140 Env-specific antibody responses were attenuated in mice that were inoculated with SmE compared to controls. Lastly, Sm-infected mice that were vaccinated with DNA+MVA displayed exacerbated liver pathology as indicated by larger granulomas and increased hepatosplenomegaly when compared with unvaccinated Sm-infected mice. This study shows that chronic schistosomiasis attenuates both HIV-specific T-cell and antibody responses and parasite elimination by chemotherapy may partially restore cellular but not antibody immunity, with additional data suggesting that the presence of SmE retained in the tissues after antihelminthic therapy contributes to lack of full immune restoration. Our data further suggest that helminthiasis may compromise HIV vaccine safety. Overall, these findings suggested a potential negative impact on future HIV vaccinations by helminthiasis in endemic areas.

## Author summary

Chronic parasitic worm infections are thought to reduce the efficacy of vaccines. Given that HIV and worm infections are common in sub-Saharan Africa (SSA) and their geographical distribution vastly overlaps, it is likely that future HIV vaccines in SSA will be administered to a large proportion of people with chronic worm infections. This study examined the impact of *S*. *mansoni* worm infections on the immunogenicity of candidate HIV vaccines in a mouse model. *S*. *mansoni* worm-infected animals had lower magnitudes of HIV vaccine responses compared with uninfected animals and elimination of worms by praziquantel treatment prior to vaccination conferred only partial restoration of normal immune responses to vaccination. The presence of *S*. *mansoni* eggs trapped in the tissues in the absence of live infection was associated with poor vaccine responses. In addition, this study found that effective immunization with some HIV vaccine regimens could potentially worsen worm-associated pathology when given to infected individuals. These novel findings suggest further research in HIV vaccines and future vaccination policies regarding the current clinical vaccines and future HIV vaccination with respect to parasitic worm infections especially in SSA.

## Introduction

Human immunodeficiency virus (HIV) and parasitic helminthic worm infections are highly prevalent and geographically overlap each other in sub Saharan Africa (SSA) [1, 2]. A majority of inhabitants harbor at least one or more species of parasitic helminth infection [3–6] and an estimated 50% of the chronically infected individuals living in high-risk rural communities are co-infected with HIV [7]. Furthermore, re-infections after successful treatments are also very common in endemic areas. Therefore, it is very likely that successful future HIV vaccines will be administered to people who already have ongoing helminthiasis or have been previously infected and treated.

Current HIV-1 vaccine research suggests that a successful HIV vaccine will need to induce effective T cell and functional antibody responses, where a key component of immune protection would be conferred through a T helper 1 (Th1) immune pathway [8, 9]. Induction of potent T cell mediated immune responses has previously been demonstrated using heterologous prime-boost vaccination strategies that utilise DNA and viral vaccine vectors such as modified Vaccinia Ankara (MVA) [10–14], while induction of durable antibody immune responses may require immunisation with HIV envelope protein-based vaccines [15–18]. It is widely accepted that an ideal HIV vaccine should induce both anti-HIV cellular responses and HIV Env-specific antibodies to destroy virus-infected cells and neutralize viruses at portals of entry respectively in order to clear the virus before dissemination into the tissues or block viral entry at the mucosal sites [8, 9, 19, 20].

During chronic schistosomiasis, parasite eggs are lodged in the liver and intestinal tissue [21, 22] resulting in predominantly T-helper 2 (Th2) immune responses [23–27] and the induction of anti-inflammatory regulatory T-cells (Treg) which suppress the innate and adaptive T- and B-cell responses [24, 28, 29]. This has been shown to lead to general hyporesponsiveness which may adversely impact standard immunizations, by suppressing immune responses to Th1-type vaccine and impairing the expansion of pathogen-specific cytotoxic T lymphocyte (CTL) responses [30–37].

Parasitic helminth infections are currently treated with chemotherapeutic drugs such as praziquantel (PZQ) for schistosomiasis [38–40] and ivermectin or mebendazole for geohelminths [41–43], which are cost-effective interventions. However, re-infection after effective treatment is common and frequent in populations in endemic areas [44]. Several animal and clinical studies have reported that helminth infections impair the outcome of a variety of vaccines, including Salmonella [45]; BCG [30, 46–48], tetanus [46, 49–51], diphtheria toxoid [52], HBV [53], pneumococcal [54] and live attenuated oral cholera vaccines [55]. However, elimination of helminth infection has also been shown to at least partially restore this abrogation [56]. Furthermore, individuals treated with antihelminthics show higher frequencies of BCG-specific IFN-γ and IL-12 producing cells than untreated helminth infected individuals [30].

Previous HIV vaccines studies reported reduced vaccine-induced immunity in schistosome-infected mice [57] and partial restoration after elimination of helminths [58, 59]. However, it is not clear if antibody responses are attenuated as these studies evaluated only cellular responses to Gag as they were monovalent candidate vaccines. Current vaccine candidates and future successful vaccines will likely include multiple immunogens, including Env, in order to broaden the vaccine targets and the capacity to cross-neutralise the majority of transmitted viruses. [60, 61].

It is well-accepted that helminth-induced Th2 responses play an important role in host protection [62, 63]. Since Th1 and Th2 display reciprocal antagonist, it would be anticipated that HIV vaccine-generated Th1 responses may reduce host immunity against helminth-associated pathology, thereby compromising the safety of an otherwise effective HIV T-cell vaccine. Poxvirus-vectored HIV vaccines are promising candidates for induction of T cell responses and therefore this is a relevant safety issue which remains under-investigated in the helminthic infection background.

We have previously described the development of two multigene candidate vaccines, the SAAVI DNA-C2 and SAAVI MVA-C, which express matched HIV-1 subtype C proteins (Gag, RT, Tat, Nef and Env) [11, 64–66]. These vaccine candidates have been evaluated further in nonhuman primates [18, 67] and Phase 1 clinical trials [15, 16]. Also, we have evaluated these vaccines in combination with an HIV-1C gp140ΔV2 Env protein [11, 15, 16, 18, 64, 65, 67].

In the current study, we investigated the impact of chronic schistosomiasis on the immunogenicity of these vaccines in a mouse model and whether the elimination of worms by antihelminthic chemotherapy prior to immunization benefits vaccination outcome. We further investigated whether the *S*. *mansoni* eggs (SmE) in the absence of active infection, which mimics the state whereby SmE remain trapped in the tissues shortly after antihelminthic treatment, has an adverse effect on vaccine immunogenicity. Lastly, we evaluated helminth-induced pathology to predict HIV vaccine safety in helminth endemic areas. Our findings show that mice infected with *S*. *mansoni* displayed reduced magnitudes of vaccine-specific cellular and humoral responses and anthelminthic treatment with PZQ failed to restore levels of anti-gp140 antibodies while partially reversing the adverse impact on cellular responses. Unexpectedly, vaccination with a T-cell based vaccine regimen was observed to worsen helminth-associated pathology suggesting potential safety concerns in future mass HIV vaccination in helminth endemic areas.

## Results

### Sm-infection impairs Th1-like cellular and humoral immunity in HIV vaccinated mice

**Fig 1:**
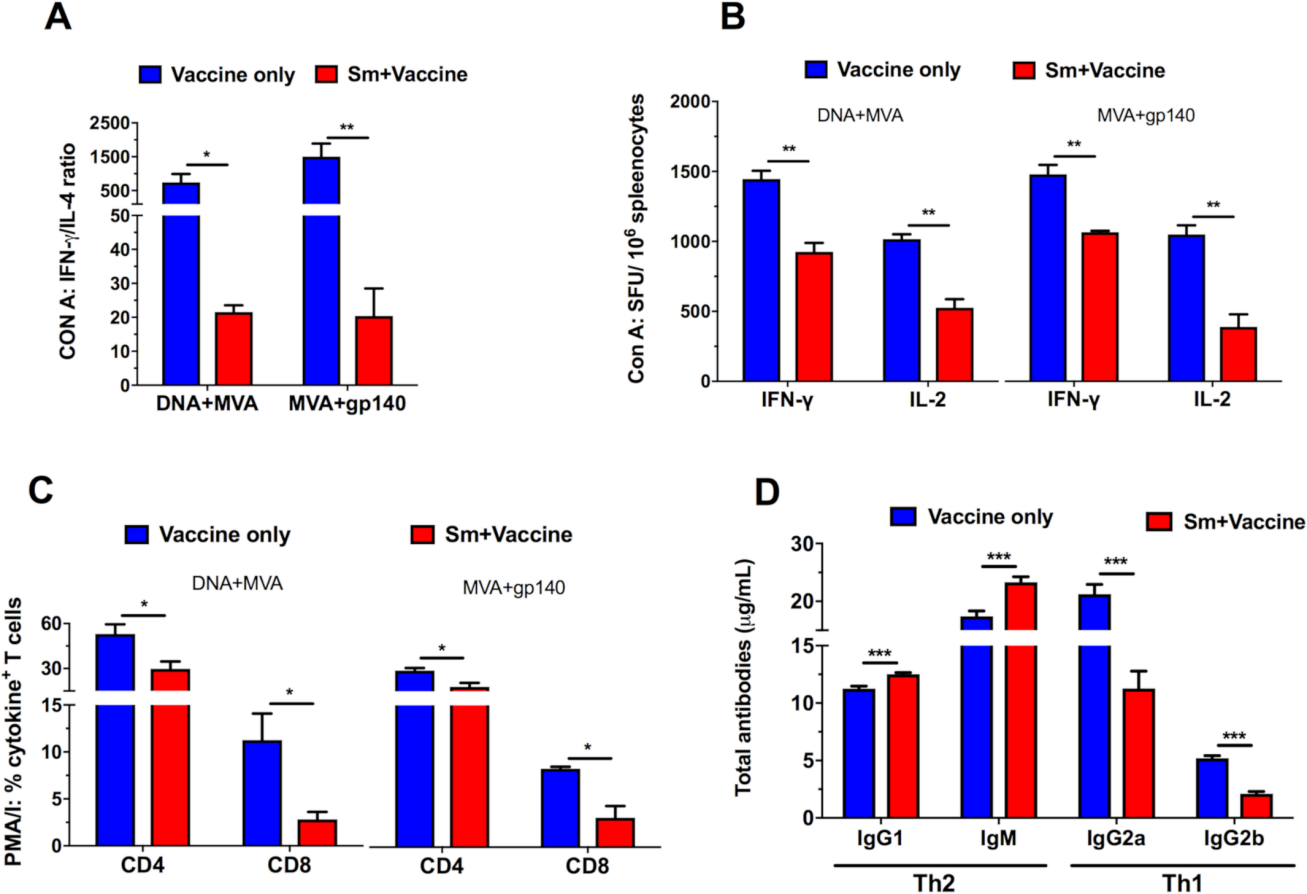
Th1/Th2 profile: *S*. *mansoni* infection induces a strong Th2 biased cellular and antibody responses. Spleens and blood were obtained from vaccinated Sm-free (blue) and Sm-infected (red) mice 16 weeks post infection. Splenocytes were prepared and stimulated with 1 μg/ml Con A for 48 (A) and 23 (B) hours and cytokines were measured in cytometric bead array and ELISpot assays respectively. Other splenocytes were stimulated with PMA/Ionomycin for 6hrs (C) and the induced intracellular cytokines were measured by flow cytometry. Total Th1 and Th2 antibodies were also measured in sera using an antibody ELISA (D). Results represent 3 independent experiments and plotted as the mean + SEM. Statistical analysis was performed using unpaired, two-tailed t-test analysis followed by FDR for multiple comparisons. (*: p<0.05; **: p<0.01; ***: p<0.001).

To assess the impact of Sm-infection on systemic immune responses, we quantified systemic Th1 and Th2 immune responses in uninfected and Sm-infected mice following MVA+gp140 and DNA+MVA vaccination. Con A stimulation of splenocytes from Sm-infected mice resulted in a significantly reduced IFN-γ:IL-4 ratio in DNA+MVA (p<0.05) and MVA+gp140 (p<0.01) vaccine regimens compared to Sm-uninfected mice (Fig 1A).

Similarly, Con A-stimulated splenocytes from Sm-infected mice produced significantly lower (p<0.05) levels of IFN-γ and IL-2 ELISpot responses compared to splenocytes from uninfected mice (Fig 1B). Furthermore, Sm-infected mice had a reduced frequency (p<0.05) of cytokine-producing CD4+ and CD8+ T cells after re-stimulation of splenocytes with PMA/*Ionomycin* compared to splenocytes from uninfected control mice (Fig 1C). Moreover, vaccinated Sm-infected mice displayed an impaired type 1 antibody response, indicated by reduced amount of type 1 total antibody isotypes [(IgG2a (p<0.001), IgG2b (p<0.001)] and increased type 2 antibody isotype [(total IgG1 (p<0.001) (Type 2-associated antibodies), IgM (p<0.001)] compared to uninfected but vaccinated mice and uninfected control mice (Fig 1D). Sm-infection was accompanied with increased levels of the regulatory cytokine IL-10 (S1C and S1I Fig).

### T cell vaccine-specific responses to SAAVI HIV-1 vaccines are attenuated by chronic helminth infection while antihelminthic treatment only partially restores the magnitudes to normal responses

**Fig 2:**
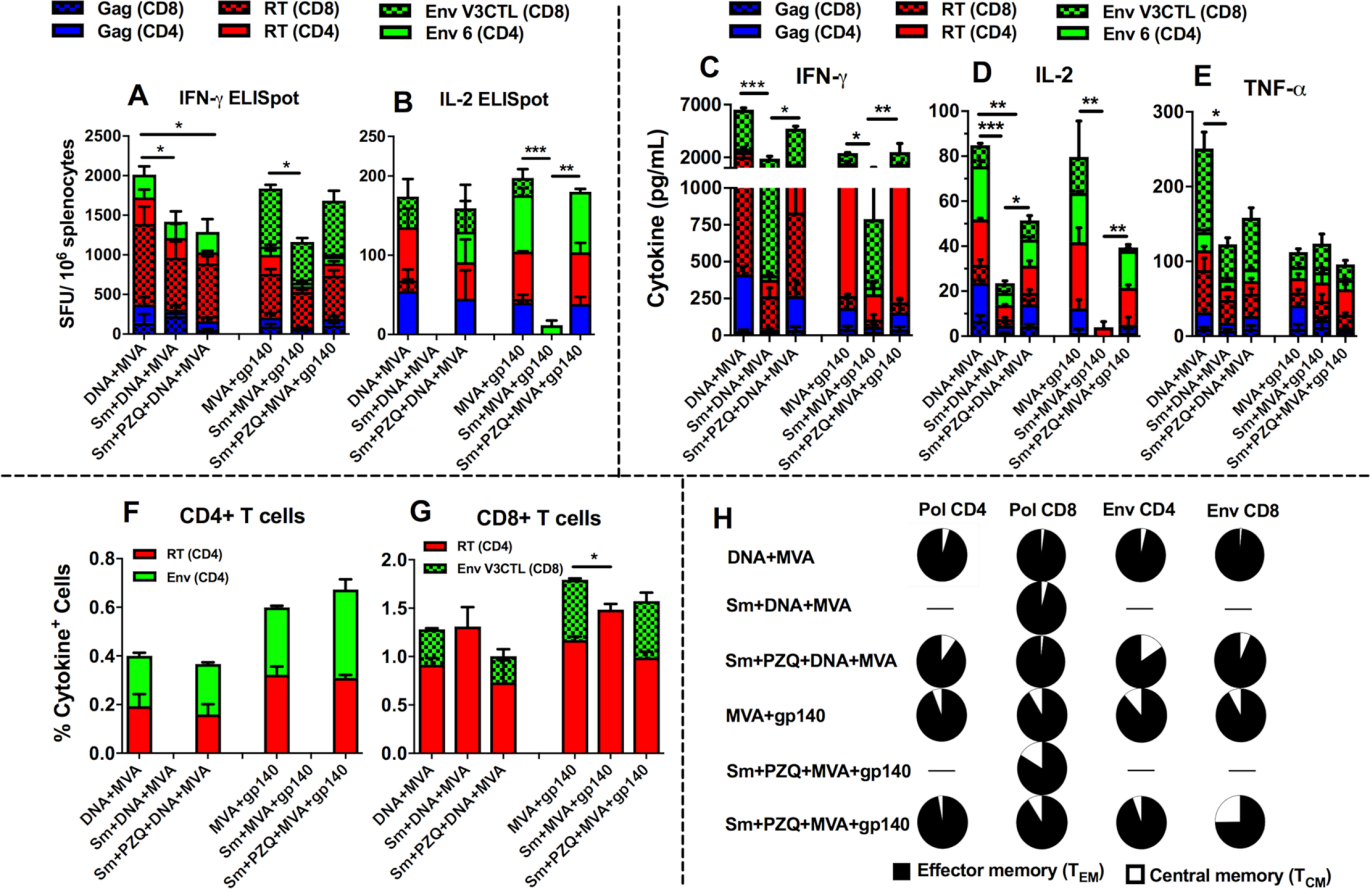
Sm infection alters vaccine-specific cellular responses and treatment with PZQ only partially restores these responses close to normal. Mice were chronically infected with Sm before vaccination with a DNA-vectored and MVA-vectored HIV-1 (DNA+MVA) vaccine or an MVA-vectored HIV-1 and a HIV-1 gp140 Env protein (MVA+gp140) vaccine regimen with or without prior anti-helminthic treatment with PZQ. Spleens were collected 12 days after the last vaccination. The splenocytes were stimulated and the induced cytokines measured in an IFN-γ (A) and IL-2 (B) ELISpot, cytometric bead array (C, D, and E), and intracellular cytokine staining (F, G, and H) assays. Results represent 3 independent experiments and plotted as the mean + SEM. Statistical analysis was performed using unpaired, two-tailed t-test analysis followed by FDR for multiple comparisons. (*: p<0.05; **: p<0.01; ***: p<0.001).

To determine the effect of chronic Sm infection on the HIV-1 vaccine-specific T cell immunity, Sm-infected and uninfected mice were vaccinated with either DNA+MVA or MVA+gp140 vaccine regimens and vaccine-specific T cell responses were determined using ELISpot, CBA and flow cytometry. To determine if elimination of schistosome infection prior to vaccination could reverse the effect on those responses, groups of mice were treated with PZQ before vaccinations. Vaccination with DNA+MVA induced significantly higher cumulative HIV-1 specific IFN-γ (2014 ± 177.4 SFU/10^6^ splenocytes) and IL-2 (174.1 ± 71.13 SFU/10^6^ splenocytes) ELISpot responses in uninfected mice compared to Sm-infected mice (IFN-γ: 1420 ± 61.54 SFU/10^6^ splenocytes and IL-2: 0 SFU/10^6^ splenocytes) (Fig 2A and 2B). Responses to the RT (CD8) peptide induced the highest number of IFN-γ secreting CD8+ and CD4+ T cells (1019 ± 217.3 SFU/10^6^ splenocytes) compared to other peptides in uninfected mice vaccinated with DNA+MVA. However, IL-2 SFU/10^6^ cells were similar among different peptides stimulations. Similarly, vaccination with MVA+gp140 induced significantly higher cumulative HIV-1 specific IFN-γ (1838 ± 173.3 SFU/10^6^ splenocytes) and IL-2 (197.7 ± 20.12 SFU/10^6^ splenocytes) ELISpot responses in uninfected mice compared to Sm-infected mice (IFN-γ: 1166 ± 132.2 SFU/10^6^ splenocytes) and IL-2: 11.89 ± 5.951 SFU/10^6^ splenocytes) (Fig 2A and 2B). Responses to the Env (CD8) peptide induced the highest number of IFN-γ secreting CD8+ and CD4+ T cells (553.3 ± 55.86 SFU/10^6^ splenocytes) compared to other peptides in uninfected mice vaccinated with MVA+gp140. However, IL-2 SFU/10^6^ cells were similar among different peptides stimulations.

Vaccination after PZQ treatment had varying effects on the magnitudes of ELISpot responses. For DNA+MVA vaccine regimen, the cumulative magnitude of IFN-γ but not IL-2 SFU/10^6^ cells was still significantly lower in treated mice compared with uninfected mice indicating partial restoration of responses to normal magnitudes (Fig 2A and 2B). In contrast, the cumulative magnitudes of both IFN-γ and IL-2 SFU/10^6^ cells between PZQ-treated and vaccinated mice and uninfected mice were similar for MVA+gp140 vaccine regimen, indicating restoration to near normal SFU/10^6^ cells (Fig 2A and 2B).

Th1 cytokine levels were significantly reduced in Sm-infected mice. As shown in Fig 2C–2E, significantly higher levels of net cumulative IFN-γ (6523 ± 282.0 pg/ml); IL-2 (84.86 ± 0.3147 pg/ml) and TNF-α (251.2 ± 30.33 pg/ml) (Fig 2C–2E) were released by splenocytes from uninfected mice in the DNA+MVA vaccine regimen compared to lower levels of IFN-γ (1899 ± 244.6 pg/ml); IL-2 (23.55 ± 4.094 pg/ml) and TNF-α (122.9 ± 17.45 pg/ml) released in Sm-infected vaccinated mice (Fig 2C–2E). Similarly, for the MVA+gp140 vaccine regimen, significantly higher levels of net cumulative Th1 cytokines: IFN-γ (2416 pg/ml); IL-2 (63.79 pg/ml) and lower TNF-α (112.48 pg/ml) were released from splenocytes of uninfected vaccinated mice compared to lower levels of IFN-γ (789 pg/ml); IL-2 (4.0 pg/ml) and higher TNF-α (123.87 pg/ml) released in Sm-infected vaccinated mice (Fig 2C–2E). After treatment with PZQ, the levels of IFN-γ and IL-2 were observed to be significantly higher compared to Sm-infected mice for both DNA+MVA and MVA+gp140 vaccine regimens but noticeably lower than those of uninfected vaccinated mice, indicating only partial restoration to normal magnitudes. Furthermore, the frequencies of vaccine-specific cytokine (IFN-γ, IL-2 and TNF-α) producing T cells as determined by flow cytometry showed a similar general trend whereby lower levels of HIV-specific T cells were detected in Sm-infected mice compared with uninfected animals (Fig 2F and 2G). For both vaccine regimens, Pol- and Env- specific CD4+ T cells were undetectable in Sm-infected vaccinated mice whilst they were readily detected at similar levels in both uninfected and PZQ-treated vaccinated mice indicating restoration of cytokine responses by PZQ treatment (Fig 2F). However, Pol- and Env- specific CD8+ T cells were detected in Sm-infected mice at similar levels as the uninfected and PZQ-treated vaccine group except in the MVA+gp140 vaccine regimen where a significantly higher percentage of cumulative cytokine-producing CD8+ T cells in response to the Pol and Env CD8 peptides stimulation was observed in uninfected vaccinated mice (1.79 ± 0.04%) compared to Sm-infected vaccinated (1.49 ± 0.06%) mice. (Fig 2G). Most of the cytokine producing CD8+ and CD4+ T cells belonged to the effector memory phenotype (Fig 2H) and the profiles of the memory phenotypes were similar in both uninfected and PZQ-treated vaccinated groups.

### Env-specific antibody responses to SAAVI MVA-C+gp140 Env vaccine regimen are attenuated by chronic helminth infection despite prior antihelminthic treatment

**Fig 3:**
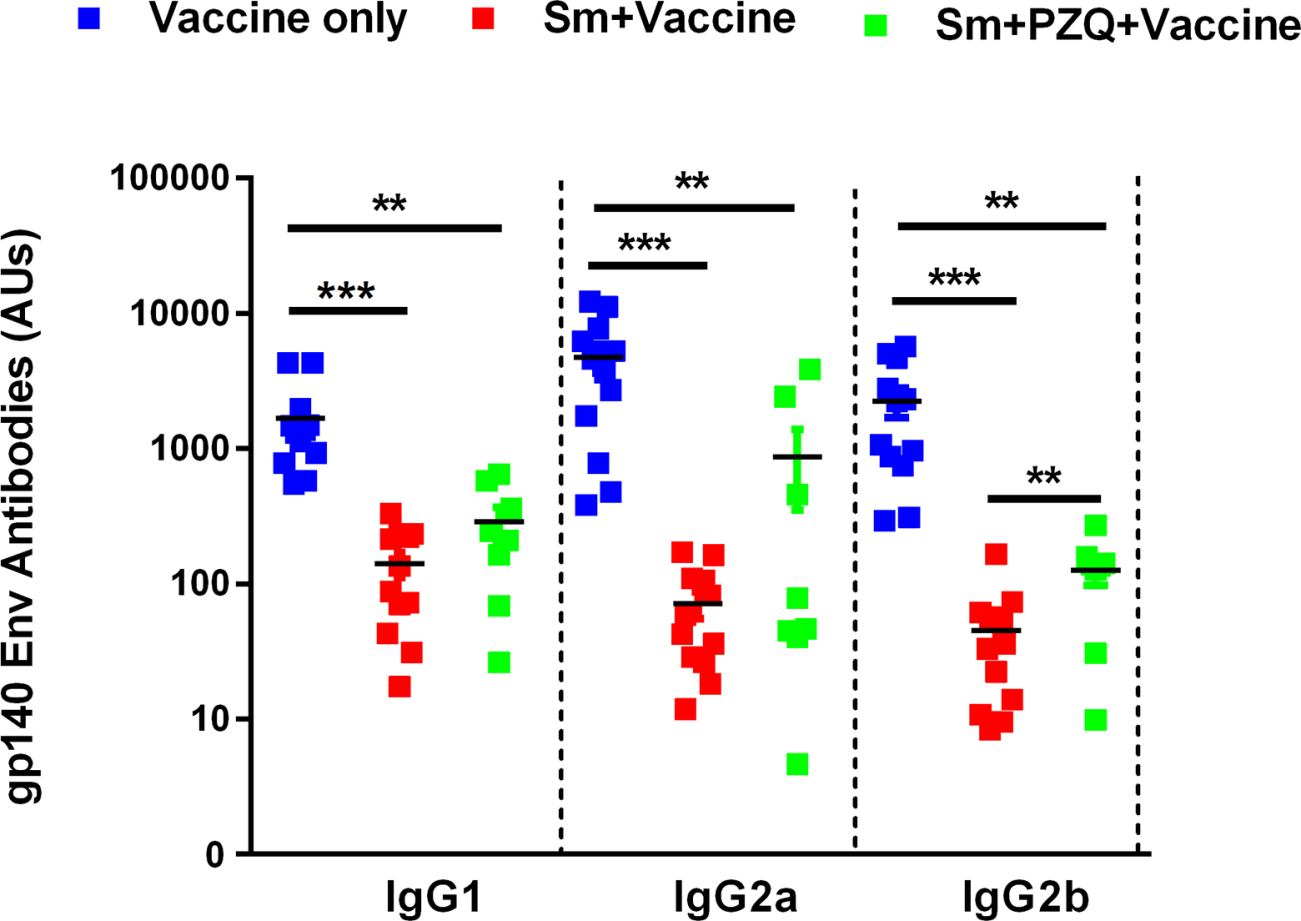
*Schistosoma mansoni* infection significantly reduces HIV-1 Env gp140-specific antibodies. Blood for preparation of serum was collected two weeks after the last vaccination. HIV-1 Env gp140-specific IgG1, IgG2a and IgG2b antibodies were analysed by ELISA. Values are plotted and expressed as mean antibody units (AUs) ± SEM for the 8–12 animals in each group. Statistical differences between the groups were calculated by unpaired t-test (two-tailed). (*: p<0.05; **: p<0.01; ***: p<0.001).

To determine the effect of helminth infection of the development of Env-specific antibody responses, mice were infected with Sm and vaccinated with MVA+gp140 and humoral responses were determined by ELISA. Uninfected and vaccinated mice produced higher amounts of gp140-specific IgG antibodies compared to Sm-infected vaccinated mice across all IgG isotypes (IgG1 [1681 ± 373.9 vs 140.8 ± 29.42 AUs]; IgG2a [4746 ± 1154 vs 71.14 ± 15.98 AUs]; IgG2b [2247 ± 553.9 vs 45.23 ± 12.65 AUs]). Treatment of infected mice with PZQ did not restore vaccine-specific antibody responses in infected mice as indicate by significantly lower titers of gp140-specific IgG antibodies across all IgG isotypes (IgG1 [287.0 ± 79.96 AUs], IgG2a [866.1 ± 514.3 AUs], IgG2b [126.3 ± 28.41 AUs]) compared to uninfected vaccinated control mice (Fig 3).

### *Schistosoma mansoni* eggs (SmE) attenuates Env-specific responses and partially suppresses T cell responses to SAAVI MVA-C+gp140 Env vaccine regimen

We next sought to investigate whether Sm eggs (SmE) alone are capable of attenuating HIV vaccine-specific responses in the absence of an active Sm infection. To achieve this, we sensitized mice with 2 500 SmE intraperitoneally, challenged them with 2 500 SmE intravenously 14 days later and vaccinated them with the MVA+gp140 vaccine regimen.

**Fig 4:**
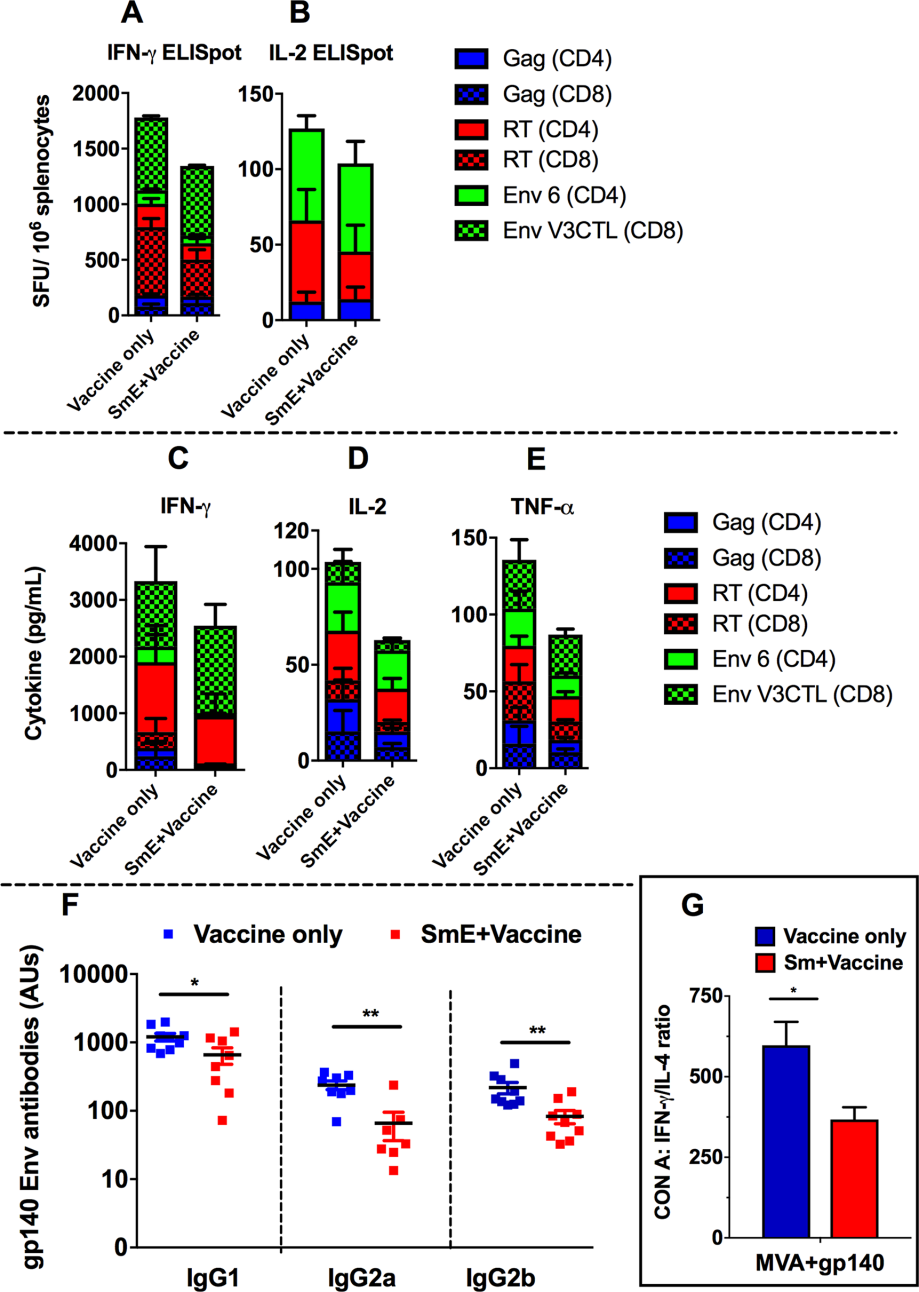
Cellular and antibody responses to HIV vaccines in SmE inoculated mice. Spleens and blood were collected 12 days after the last vaccination. Splenocytes were stimulated the induced cytokines were analysed in an IFN-γ (A) and IL-2 (B) ELISpot, cytokine bead array (CBA) (C, D, and E) assays. The individual bars represent the magnitudes of the cumulative cytokine levels. HIV-1 Env gp140-specific IgG1, IgG2a and IgG2b antibodies were analysed in the sera by an ELISA (F). Values are expressed as antibody units and the mean (AUs) for the 8–12 animals in each group shown as a horizontal bar. An IFN-γ:IL-4 ratio (G) was calculated from the CBA data. Results represent 3 independent experiments. Statistical analysis was performed using unpaired, two-tailed t-test analysis. (*: p<0.05; **: p<0.01).

Cumulative cellular responses to the HIV peptides were measured in the spleens using CBA and ELISpot (Fig 4A–4E) and gp140 Env-specific antibodies in the sera (Fig 4F). Cumulative HIV-1 IFN-γ SFU/10^6^ (Fig 4A), and IL-2 SFU/10^6^ (Fig 4B) in SmE-inoculated vaccinated mice were noticeably lower, but not significantly when compared to SmE-free vaccinated mice. Similarly, levels of cumulative IFN-γ; TNF-α and IL-2 secreted by splenocytes were noticeably lower in SmE-inoculated vaccinated mice compared to those secreted by splenocytes from SmE-free vaccinated mice (Fig 4C–4E respectively). However, SmE-inoculated mice had significantly lower amounts of gp140-specific IgG1 (656.8 ± 177.1 versus 1203 ± 152.0 AUs), IgG2a (71.14 ± 15.98 versus 238.1 ± 34.33 AUs), and IgG2b antibodies (82.73 ± 18.20 versus 218.3 ± 41.86 AUs) compared to SmE-free mice (Fig 4F), indicating broad attenuation of gp140 Env-specific antibody responses. Furthermore, we confirmed that SmE alone, in the absence of active infection, is capable of skewing the Th1/Th2 profile towards a Th2 response. As shown in Fig 4G, at 9 weeks post inoculation, the IFN-γ:IL-4 ratio was significantly lower in vaccinated SmE-inoculated (367.0 ± 38.34 pg/ml) compared to SmE-free (597.2 ± 72.93 pg/ml) vaccinated mice after stimulation with Con A. Similarly, stimulation of splenocytes with SEA resulted in a trend towards reduced IFN-γ:IL-4 ratio for SmE-inoculated mice compared to uninfected but vaccinated mice (S2 Fig).

### Vaccination with DNA+MVA vaccine regimen worsens the Sm-associated pathology in mice

**Fig 5:**
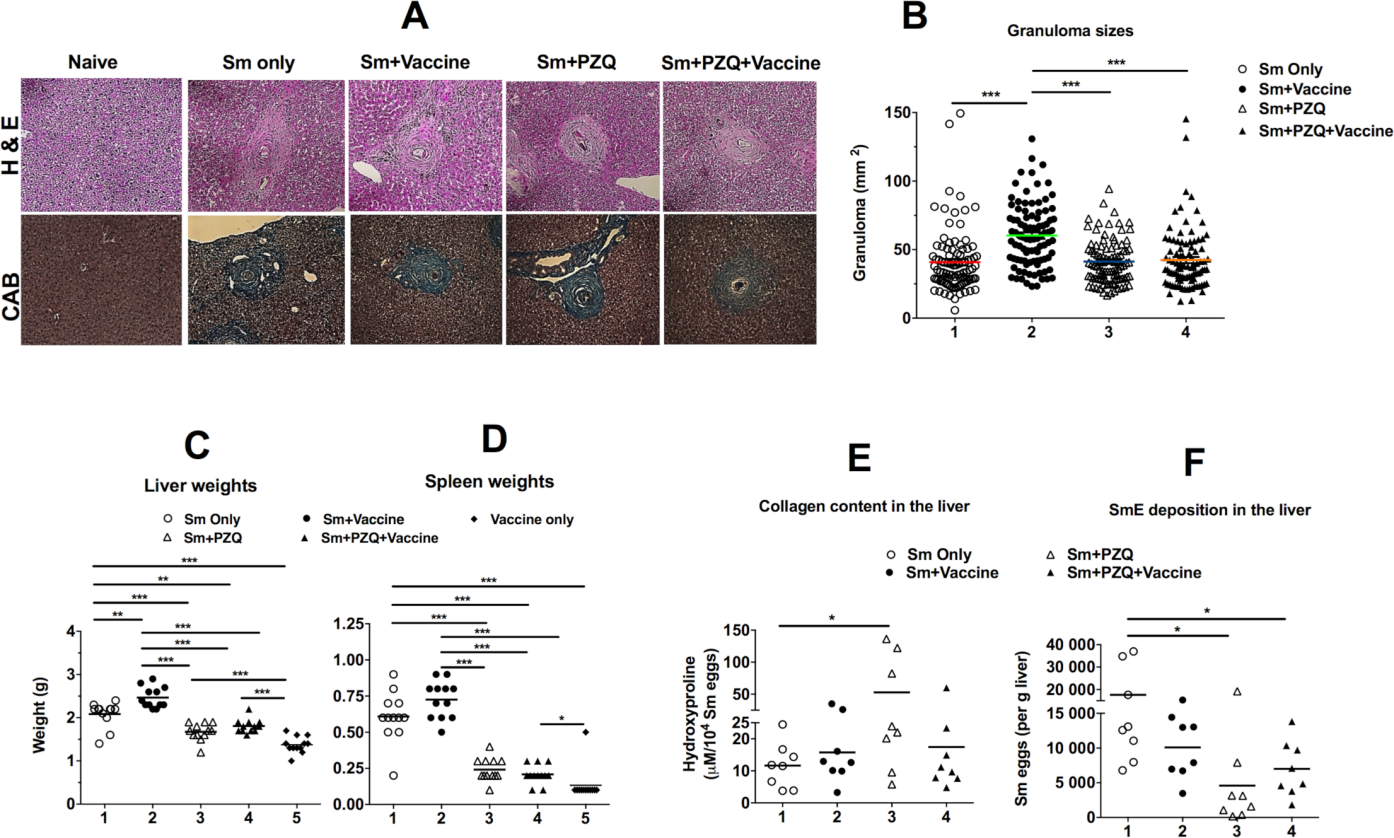
Analysis of livers and spleens of mice after vaccination with a DNA+MVA vaccine regimen. Groups of mice were infected with 30–35 Sm cercariae. Some groups were treated twice with PZQ after 8.5 weeks post infection. Vaccination with a DNA+MVA vaccine regimen was performed as shown in Table 1. Spleens and livers were harvested 12 days after last vaccination, weighed and prepared for various analyses. (A): Representative histological micrographs showing liver granulomas after staining with either H&E or CAB; (B): Granuloma sizes; (C) Liver weights; (D): Spleen weights; (E): hydroxyproline content and (F): Number of eggs per gram of liver. For Figs B-D, the results represent 3 independent experiments. For Figs E and F, the data is representative of 2 independent experiments. Means are shown as horizontal bars. Statistical analysis was performed using unpaired, two-tailed t-test analysis followed by FDR for multiple comparisons. (*: p>0.05; **: p<0.01; ***: p<0.001).

We investigated whether vaccination with DNA+MVA exacerbates helminth associated pathology in chronically infected mice by determining granuloma sizes and hydroxyproline content in mouse livers and assessing hepatosplenomegaly. We also investigated whether treatment with PZQ prior to vaccination with the DNA+MVA regimen ameliorates tissue pathology in infected mice. Sm-infected and vaccinated mice developed significantly larger (60.27 ± 2.37 mm^2^) granulomas when compared to all the other groups (vaccinated Sm-infected-PZQ treated [41.29 ± 1.66 mm^2^]; Sm-infected alone [40.79 ± 2.38 mm^2^] and Sm-infected-PZQ treated vaccinated [42.36 ± 2.26 mm^2^]) (Fig 5A and 5B). Sm-infected mice that were either vaccinated or unvaccinated developed hepatosplenomegaly as indicated by larger spleens and livers compared to vaccinated infected mice, naïve mice and vaccinated Sm-infected-PZQ treated mice (Fig 5C and 5D). No difference in hydroxyproline content was observed between unvaccinated Sm-infected and vaccinated Sm-infected mice (Fig 5E). Surprisingly, high levels of hydroxyproline content were observed in unvaccinated Sm-infected-PZQ treated mice compared to Sm-infected (Fig 5E). However, Sm-infected mice had significantly higher number of eggs per gram of liver compared to unvaccinated and vaccinated Sm-infected mice that were treated with PZQ (Fig 5F).

## Discussion

The present study investigated the impact of chronic schistosomiasis on the induction of T cell-mediated and antibody responses to candidate HIV vaccines in a mouse model, whether attenuation of vaccine responses can be reversed by pre-vaccination anti-helminthic treatment, and if vaccination with a T cell-based candidate vaccine has an adverse effect on helminth-associated pathology.

Firstly, we sought to establish that the mouse model of chronic schistosomiasis worked well as to be expected on our hands. As we expected, our data confirmed that prior to vaccination, Sm-infected mice elicited predominantly Th2 responses and a decreased Th1 cytokine profile (Fig 1A and 1B; S1 Fig), had impaired Th1 cytokine-producing CD8+ and CD4+ T cells (Fig 1C) and an increase in Th2 total antibodies in serum (Fig 1D) as well as enlarged spleens and livers (Fig 5C and 5D) compared to uninfected mice. These findings are consistent with previous reports that demonstrate that Sm infection and a host of other helminths skews the host’s immune responses from a Th1 towards a Th2 type with egg deposition and increased production of IL-4 as key driving forces [23, 26, 27, 63, 68–72]. Our data also agrees with previous studies that have demonstrated that IL-10 is also responsible for down-regulation of Th1 responses that is observed in schistosome infections [73, 74]. IL-10 has been shown to mediate this down-regulation via an activation-induced cell death process resulting in apoptosis of CD4+ and CD8+ T cells which is also linked to the onset of egg-laying by the helminth parasite and formation of granulomas [75–77]. Our data also suggested a correlation between the decrease of cytokine-producing CD8+ T cells and levels of IgG2a and IgG2b antibodies observed in unvaccinated Sm and vaccinated Sm-infected mice. Previous studies have reported that cytokines produced by CD4+ and CD8+ T cells play an important role in the regulation of the humoral immune response and isotype switching [78–80]. The immunological consequence of a predominant Th2 biasing demonstrated in Sm-infected mice agrees with the concept of reciprocal antagonism between Th1 and Th2 as previously suggested [62, 81].

Secretion of IFN-γ and IL-2 by T cells has been associated with suppression of viral replication in HIV-infected individuals and better proliferation of HIV-1-specific CD4+ and CD8+ T cells, suggesting that production of these Th1 cytokines by candidate HIV vaccines is a good indicator of vaccine-mediated immune protection [82] and good indications of polyfunctional CD4+ T cell responses [83]. In this study, we observed that the presence of Sm infection prevented optimal generation of vaccine-specific T cell responses following immunization with SAAVI vaccine candidates (Fig 2). As shown in Fig 2C–2E, vaccine-specific Th1 cumulative cytokine levels (IFN-γ, IL-2 and TNF-α) in recall responses to HIV peptides were significantly reduced in vaccinated Sm-infected compared to Sm-free mice vaccinated with DNA+MVA and MVA+gp140, with exception to TNF-α in MVA+gp140 vaccinated groups which were similar in both vaccinated Sm-infected and Sm-uninfected but vaccinated mice. Similarly, the magnitudes of vaccine-specific cumulative IFN-γ and IL-2 T cell responses measured by ELISpot were significantly lower in Sm-infected vaccinated mice compared to Sm-uninfected mice vaccinated with DNA+MVA and MVA+gp140 (Fig 2A and 2B). A similar decline has been reported in Sm-infected mice compared to uninfected controls following immunization with a DNA-vectored HIV-1 vaccine [57]. Also, the frequencies of vaccine-specific cytokine-producing CD4+ and CD8+ T cells in Sm-infected mice were observed in the current study (Fig 2F and 2G), which may translate to a decrease in antibody population [84]. However, it was noted that the cytokine-producing T cells were predominantly of the effector memory phenotype in both Sm-infected and uninfected vaccinated mice (Fig 2H). Vaccine-induced effector memory T cells have been associated with protection against mucosal SIV challenge in vaccinated rhesus monkeys [85]. In this study, the downregulation of these vaccine-specific cellular responses demonstrates the ability of Sm infection to negatively affect protective potential of candidate HIV-1 vaccines as have suggested by others [57, 59].

The antibody responses to the gp140-Env protein were significantly impaired in Sm-infected vaccinated mice (Fig 3). The mean concentration of anti-gp140 antibodies in Sm-infected mice vaccinated with MVA+gp140 was significantly lower than that in Sm-free vaccinated control for all IgG isotypes (IgG1; IgG2 and IgG2b). Antibody responses to specific HIV antigens have been proposed to correlate with protection [82, 86]; thus, this was an interesting finding with far-reaching implications for future vaccine development.

Elimination of helminth parasites with an antihelminth drug prior to immunization was expected to restore normal vaccine T cell responsiveness as previously demonstrated [59, 87, 88]. Our results show that treating mice with PZQ reversed tissue pathology as indicated by reduced spleen and liver weights sizes of granulomas and SmE deposition in the liver tissue in PZQ-treated mice compared with untreated mice (Fig 5B–5D and 5F) is consistent with earlier reports [89, 90]. Treatment with PZQ has been shown to eliminate adult worms with no direct impact on the SmE already trapped in the tissues other than preventing continued egg deposition in treated subjects [38–40] and potentially restoring normal T cell immune responsiveness. However, our immunological data showed that treating mice prior to vaccination only partially restored the hosts’ vaccine-specific T cells responses (Fig 2). Surprisingly, the partial recovery of these responses did not translate in reduction of the magnitudes of Th2 cytokine responses (S1 Fig). Anti-inflammatory cytokines such as IL-10 remained elevated despite treatment with antihelminth (S1C and S1I Fig) whilst previous studies in which PZQ was used reported similar findings [58, 59]. However, it was unclear if the antibody responses were affected. In our study, Th1-type gp140-Env-specific antibody responses in Sm-infected mice were significantly lower despite treatment with PZQ (Fig 3). To our knowledge, no study has evaluated the ability of antihelminthic treatment in the restoration of antibody responses to HIV vaccines. However, it is has been suggested that the duration of infection prior and post treatment is an important factor which determines subsequent restoration of normal responses to vaccination [53, 89, 90]. A study by Chen et al., showed a recovery of immune balance 16 weeks post-treatment [53]. Also, findings from the studies conducted by Da’dara’s group and Shollenberger’s groups, demonstrated that normal immune responses can be achieved 2–10 weeks post-treatment [58, 59]. In contrast to our study, only a 1.5-week post-treatment period was allowed prior to commencing the vaccinations. Future studies should investigate varying post-treatment periods including multiple vaccinations to establish the optimal recovery durations to start vaccinations after antihelminthic treatment. This is particularly relevant if there will arise a need to integrate future HIV vaccinations with helminthic worms control programmes to improve the vaccination outcomes in helminth-endemic areas.

This study went further to demonstrate that Th1 cellular responses elicited by DNA- and MVA- vectored HIV-1 vaccines exacerbated helminth-induced pathology. Sm-infected mice vaccinated with a DNA+MVA regimen had significantly larger granulomas as well as enlarged spleens and livers compared to Sm-infected unvaccinated groups (Fig 5B and 5C). Treatment significantly reduced the pathology; however, a considerable number of eggs were still present in the liver tissues of treated mice (Fig 5F). Surprisingly, the amount of hydroxyproline content, which is a measure of collagen content, was significantly higher in PZQ-treated uninfected mice compared with unvaccinated Sm-infected groups (Fig 5E), suggesting that PZQ treatment may contribute to increased fibrosis of the liver (Fig 5E) as an adverse side effect. A recent study showed that a novel experimental drug (Paeoniflorin) used for treating schistosomiasis managed to control sclerosis better than PZQ [91], pointing to a possible future replacement of PZQ as the antihelminthic drug of choice. Nevertheless, PZQ treatment resulted in reduced number of eggs per gram of liver tissue when compared with Sm-infected untreated mice (Fig 5F). These findings highlighted the scientific challenges in the development of HIV vaccines for SSA, where parasitic helminthiasis is endemic.

As discussed above, this study found lack of restoration of vaccine-specific responses upon PZQ-treatment prior to vaccinations while a substantial level of SmE burden was observed in PZQ-treated mice several weeks post-treatment. We, therefore investigated if Sm eggs in the absence of a live infection could result in downregulation of HIV-specific responses. Following an established Sm-egg model [92], mice were inoculated with *S*. *mansoni* eggs and then vaccinated with candidate HIV vaccines to evaluate how these eggs affect vaccination outcomes.

The IFN-γ:IL-4 ratio for the SmE-sensitized vaccinated mice was significantly smaller than the unsensitized vaccinated mice (Fig 4G), indicating a considerable elevation of Th2 cytokines and down regulation of Th1 in SmE-inoculated mice comparable to SmE-unsensitized mice. However, this polarized Th2 immune responses appear to have had only partial effects on the vaccine-specific T cell responses (Fig 4A–4E). Although reduced, the decrease in vaccine-specific cellular responses observed in SmE-inoculated mice was not significant. Surprisingly as with Sm live infection, antibody responses to HIV Env-gp140 were significantly reduced in the presence of SmE (Fig 4F). As suggested previously [72], this finding confirms that SmE trapped in the tissues play a critical role in attenuating the host’s vaccine-specific responses in Sm-infected individual and may explain why both cellular and antibody responses are still suppressed despite treatment in PZQ-treated groups. Thus, the possible mechanism by which Sm infection suppresses these HIV-specific cellular and humoral responses appear to involve the deposition of SmE in the tissues, which stimulates increased production of IL-4 and IL-10 with concomitant polarization of Th2 immune responses. This in turn may promote activation-induced apoptosis of HIV-specific CD4+ and CD8+ T cells resulting in attenuated induction of Th1 immune responses which are key components of HIV vaccine-specific responses. This finding further highlights another challenge that even after antihelminth treatment with PZQ, generation of optimal vaccine responses may not be achieved as helminth eggs left trapped in the tissues could still attenuate HIV vaccine-induced immune responses. In light of these findings, this study suggests that, whilst elimination of worms can offer an affordable and a simple means of antihelminthic treatment, only partially restoration of immune responsiveness to T cell-based vaccines for HIV-1 and other infectious diseases in helminth endemic settings may be achieved. Thus, it would be important to evaluate vaccine delivery systems that can potentially overcome the negative impact of concurrent helminthiasis as previously suggested [93]. An alternative avenue would be the discovery of antihelminthic drugs which are effective in elimination of SmE from the host’s tissues in addition to the elimination of the parasitic worms.

Although, this study gives further information on the impact of helminth infection on the immunogenicity of HIV vaccines, not all immunological aspects could be elucidated. Thus, this study justifies further investigations with use of a nonhuman primate model such as baboons (immune system is highly similar to humans) to obtain a better understanding of these immune responses.

The present study demonstrated that chronic helminth infection is associated with Th2-driven attenuation of both T cell and antibody response to HIV vaccines, and elimination of worm by chemotherapy partially restored T cell responses but not necessarily antibody responses. This study further demonstrated that vaccinating helminth-infected individuals with HIV vaccines that induce strong cellular responses may increase the pathology induced by the parasites, rendering the vaccine unsafe in helminth endemic areas. Lastly, this study suggests that the often-suggested integration of antihelminthic treatment programme with a successful future HIV vaccine might not result in improved vaccination outcome unless alternative antihelminthic drugs with a capacity to eliminate schistosome eggs from tissues are developed. In addition, we recommend that HIV vaccine development programs should consider designing vaccines that can overcome the adverse effects of helminth-induced immunity.

## Methods

### Parasites and vaccines

*Biomphalaria glabrata* snails (Strain NMRI, NR-21962), infected with *Schistosoma mansoni* (Strain NMRI) were provided by the Schistosome Research Reagent Resource Center (NIAID, NIH, USA) and maintained in our laboratory for generation of live *S*. *mansoni* (Sm) cercariae that were used in this study. *S*. *mansoni* eggs (SmE) were purchased from the Theodor Bilharz Research Institute (Schistosome Biological Supply Center, Egypt) and stored at -80°C until use. The integrity and viability of the eggs were evaluated using a light microscope prior to use.

### Vaccines

I SAAVI DNA‐C2 (DNA): Composed of two DNA plasmids, expressing a human immunodeficiency virus subtype C (HIV-1C) polyprotein comprising *gag*, *reverse transcriptase*, *tat* and *nef* (grttnC) and an HIV-1C truncated *env* (gp150CT) as previously described [65].
II SAAVI MVA‐C (MVA): Recombinant MVA expressing the same immunogens as the DNA vaccine as previously described [64].

Both DNA- and MVA-vectored HIV-1 vaccines have been shown to elicit strong T cell responses in mice [11], nonhuman primates [18, 67] and clinical trials [16].

III HIV-1 gp140 Env protein (gp140 Env): The gp140 (TV-1) (HIV-1/Clade C) was purchased from Immune Technology, USA. The Env amino acid consequences were derived from a South African HIV-1 subtype C primary isolate, TV1 [94].

### Ethics statement

Female BALB/c mice (6–8 weeks old) were purchased from South African Vaccine Producers (SAVP) (Johannesburg, South Africa), housed in an Animal Biosafety Level 2 facility at the University of Cape Town and maintained in accordance with the South African National Guidelines for Use of Animals for Scientific Purposes (SANS Code 10386: 2008) which is also in line with EU Directive 2010/63/EU. Experimental protocols performed in this study were reviewed and approved by the Animal Ethics Committee of the University of Cape Town (UCT AEC: protocol number: 014/026) and performed by qualified personnel in compliance with the South African Veterinary Council regulations. A mixture of ketamine hydrochloride and xylazine was used to anaesthesise mice for all procedures that involved intramuscular or intravenous injections, infection with live Schistosoma mansoni cercariae, collection of blood by cardiac puncture and preparation for euthanasia. Euthanasia was done by cervical dislocation while the animals were under anaesthesia (induced with a mixture of ketamine and xylazine as describe above).

### Infection, inoculation with SmE, PZQ treatment and HIV immunization

The study comprised of three experiments. In Experiment 1 and 2 (Table 1), mice were randomly allocated to six groups (5–8 mice per group) per experiment. Mice receiving exposure to Sm (4 groups) were infected percutaneously via the abdomen with 35 live *S*. *mansoni* cercariae at the beginning of the experimentation. Those receiving antihelminthic treatment were given two doses of PZQ (Sigma Aldrich, USA) by oral gavage (500 mg/kg; diluted in water containing 2% Kolliphor EL [Sigma Aldrich, USA]) three days apart, between 8 and 8.5 weeks post infection. Animals were vaccinated twice, 4 weeks apart, starting at 10 weeks post infection (Table 1). In Experiment 3 (Table 2), mice were allocated to 4 groups (5 mice per group). Two groups were inoculated twice with SmE (2500 eggs per mouse), 14 days apart, initially by intraperitoneal route, and subsequently by intravenous route. As in Experiments 1 and 2, mice were vaccinated twice, 4 weeks apart, starting at 1 week after the second inoculation with SmE (Table 2).

**Table 1:** Immunasation schedule for live *S*. *mansoni* cercaria infection groups.

	Groups: n = 5–8	Infection	PZQ 1	PZQ 2	Vaccine 1	Vaccine 2	Sampling
Week		0	8	8.5	10	14	16
**Experiment 1**	MVA+Gp140	-	-	-	✓	✓	✓
	Sm+MVA+Gp140	✓	-	-	✓	✓	✓
	Sm+PZQ+MVA+Gp140	✓	✓	✓	✓	✓	✓
	Sm+PZQ	✓	✓	✓	-	-	✓
	Sm	✓	-	-	-	-	✓
	Naïve	-	-	-	-	-	✓
**Experiment 2**	DNA+MVA	-	-	-	✓	✓	✓
	Sm+DNA+MVA	✓	-	-	✓	✓	✓
	Sm+PZQ+DNA+MVA	✓	✓	✓	✓	✓	✓
	Sm+PZQ	✓	✓	✓	-	-	✓
	Sm	✓	-	-	-	-	✓
	Naïve	-	-	-	-	-	✓

**Table 2:** Immunisations schedule for *S*. *mansoni* egg challenge groups.

	Groups: n = 5	i.p. inoculation with 2500 SmE	i.v. inoculation with 2500 SmE	Vaccine 1	Vaccine 2	Sampling
Week		0	2	3	7	9
**Experiment 3**	MVA+gp140	-	-	✓	✓	✓
	SmE+MVA+gp140	✓	✓	✓	✓	✓
	SmE	✓	✓	-	-	✓
	Naïve	-	-	-	-	✓

Vaccinations with DNA-vectored (100μg DNA per mouse) and MVA-vectored (10^6^ plaque forming units per mouse) HIV-1 vaccines were given intramuscularly. Vaccination with HIV-1 gp140 Env (10μg protein per mouse formulated in Imject Alum adjuvant [Thermo Scientific, USA]) was administered subcutaneously. DNA+MVA vaccine regimens were given as DNA prime and MVA boost vaccine regimens whilst MVA+gp140 Env were given concurrently. Twelve days following the last vaccination, blood was collected by cardiac punctured, mice were euthanised and spleens and livers were harvested for evaluation of HIV immune responses and helminth-induced pathology.

### Immunogenicity assays to evaluate host immune response and SAAVI vaccines specific responses cellular immune response to HIV vaccines

Splenocytes were prepared using a standard protocol [95] and stimulated with HIV peptides or mitogen stimulant at 2μg/ml (S1 Table). IFN-γ and IL-2 ELISpot assays were carried out as previously described [11]. Cytometric bead array (CBA) assays were carried out as previously described [96]. Intracellular cytokine staining (ICS) and flow cytometry analysis was performed as previously described [12, 14] with minor modifications. Briefly, cells were stained with a viability dye, violet amine reactive dye (ViViD; Invitrogen, USA), at a pre-determined optimal concentration before staining for cell surface molecules with the following fluorochrome-conjugated antibodies: anti-CD3-Alexa 700, anti-CD4-PE-Cy7, anti-αCD8-APC-Cy7, anti-CD62L-APC, and anti-CD44-FITC diluted to 0.2μg in staining buffer (BD Biosciences, USA). Further intracellular cytokine staining was done with pooled PE-conjugated anti-TNF (0.2μg) anti-IL-2 (0.06μg) and anti-IFN-γ (0.06μg) antibodies diluted in Perm/Wash buffer (BD Biosciences, USA).

### Antibody responses to HIV vaccines

To measure the level of HIV gp140 Env-specific antibodies in mouse sera, a standardised ELISA assay was established as previously described [97]. Briefly, ELISA plates were coated with 0.5μg/ml of gp140 protein diluted in PBS and incubated overnight at 4°C. Test mouse sera (diluted 1:1000) were tested in duplicates. Mouse sera obtained from unvaccinated mice was used as a negative control while a reference serum sample prepared from mice previously vaccinated with HIV gp140 protein was used in 12 two-fold dilutions, starting at 1:100, to generate a standard curve.

For detection, appropriate secondary anti-mouse antibodies conjugated with horseradish peroxidase were used including the three anti-mouse IgG isotypes (IgG1; IgG2a and IgG2b; Southern Biotechnology). After colour development using tetramethyl-benzidine substrate (TMB; KPL, USA), the optical density (OD) was measured at 450nm (with a reference filter set at 540 nm) using a microplate reader (Molecular Devices Corporation, USA). Based on the constants of the standard curve generated from the serially diluted reference sample, the reciprocal dilution giving an OD value of 1 (against gp140) was assigned a value of 1000 antibody units (AUs). The negative control (unvaccinated mouse serum) was assigned a reciprocal dilution of 0 and zero AUs. A reference sample was used on each ELISA plate to generate a standard curve from which the assigned AUs were used to extrapolate for test samples at a fixed dilution of 1:1000. A cut-off value for positive antibody responses was set at 2 x the OD value of the negative control serum (unvaccinated) and those below the cut of value were assigned an antibody unit of zero.

### Histology

Spleens and livers were weighed prior to processing for immunological evaluation in the laboratory to determine if HIV vaccination worsens helminth-associated pathology. Livers were then fixed in 4% (v/v) buffered formalin solution. The fixed samples were then embedded in wax and processed. Sections (5–7μm) were stained with hematoxylin and eosin (H&E) (Sigma Aldrich, USA) to show aggregation of white blood cells around the Sm eggs and Chromotrop-aniline blue solution (CAB) (Sigma Aldrich, USA) and counterstained with Weigert's hematoxylin (Sigma Aldrich, USA) to stain for collagen. Micrographs of liver granuloma were captured using a Nikon 90i wide-field microscope using a 5.0 megapixel colour digital camera running Nikon’s NIS-Elements v. 4.30 software (Nikon Instruments Inc., USA). The area of each granuloma containing a single egg was measured with the ImageJ 1.34 software (National Institutes of Health, USA). A total of 25–30 granulomas per slide per mouse were included in the analyses. Data was presented as a mean area of each granuloma containing a single egg. The number of eggs per gram of liver was determined by counting individual eggs from hydrolysed liver under a microscope.

### Hydroxyproline assay

Hydroxyproline content, which is a direct measure of collagen content in liver was determined using a modified hydroxyproline protocol by Bergman and Loxley [98]. Briefly, liver samples were weighed, hydrolyzed and added to a 40mg Dowex/Norit mixture. The supernatants were neutralised with 1% phenolphthalein and titrated against 10 M NaOH. An aliquot was mixed with isopropanol and added to chloramine-T/citrate buffer solution (pH 6.5). Erlich’s reagent (95% ethanol containing dimethylaminobenzaldehyde (DMAB) and concentrated hydrochloric acid) was added and absorbance was read at 570 nm. Hydroxyproline levels were calculated using 4-hydroxy-L-proline (Sigma Aldrich, USA) as a standard, and results were expressed as μmoles hydroxyproline per weight of tissue that contained 10^4^ eggs.

### Statistical analysis

Statistical analysis was performed using Prism version 5.0 (GraphPad Software, USA). The t-test for independent unpaired non-parametric comparisons was applied to assess the level of significance between means ±SEM. Three independent experiments were conducted and all tests were two-tailed. p values <0.05 were considered as significant. The false discovery rate (FDR) with Benjamini-Hochberg-adjusted p<0.05 was performed as previously described [99].

## Supporting information

S1 Fig Cytokine production after Con A and SEA stimulation in Sm-free and Sm-infected mice vaccinated with MVA+gp140 or DNA+MVA regimen. Splenocytes were harvested from mice vaccinated with the indicated regimen described in Fig 1. They were then stimulated with an irrelevant peptide (negative control), Con A or with SEA for 48 hours. Culture supernatants were collected and the level of Th1 and Th2 cytokines released into the medium for MVA+gp140 (A-C and D-F respectively) and DNA+MVA (G-I and J-L respectively) vaccinated mice was measured using a cytokine bead array assay. The individual bars represent the magnitude of the net cytokine levels for vaccinated Sm-free (blue); vaccinated Sm-infected (red) and Sm-infected-PZQ treated (green) vaccinated mice. Results represent 3 independent experiments and plotted as the mean + SEM, and cytokine levels were expressed as pg/ml. Statistical analysis was performed using unpaired, two-tailed t-test analysis followed by FDR for multiple comparisons. (*: p>0.05; **: p<0.01; ***: p<0.001).
(TIF)

S2 Fig Th1/Th2 profile: The presence of *S. mansoni* Eggs (SmE) in the tissues tends to polarize the Th1/Th2 balance towards a Th2 profile. Splenocytes were obtained from SmE-sensitized and non-sensitized mice after two vaccinations with MVA-vectored HIV-1 and HIV-1 gp140 Env protein vaccines as shown in Table 2. They were then stimulated with an irrelevant peptide (negative control) or with SEA for 48 hours. Culture supernatants were collected and the level of Th1 and Th2 cytokines released into the medium was measured using a cytokine bead array assay. The individual bars represent the IFN-γ/IL-4 ratio for vaccinated non-sensitized (blue) and vaccinated SmE-sensitized (red) mice. Results represent 3 independent experiments and plotted as the mean + SEM.
(TIF)

S1 Table Control and peptide stimulants used in the ELISpot, ICS and CBA assays. (DOCX)
