# Supplementary figures and images for: Chronic schistosomiasis suppresses HIV-specific responses to DNA-MVA and MVA-gp140 Env vaccine regimens despite antihelminthic treatment and increases helminth-associated pathology in a mouse model
Source: PLoS Pathog. 2018 Jul 26;14(7):e1007182. doi: 10.1371/journal.ppat.1007182 (PMC6080792; doi:10.1371/journal.ppat.1007182)

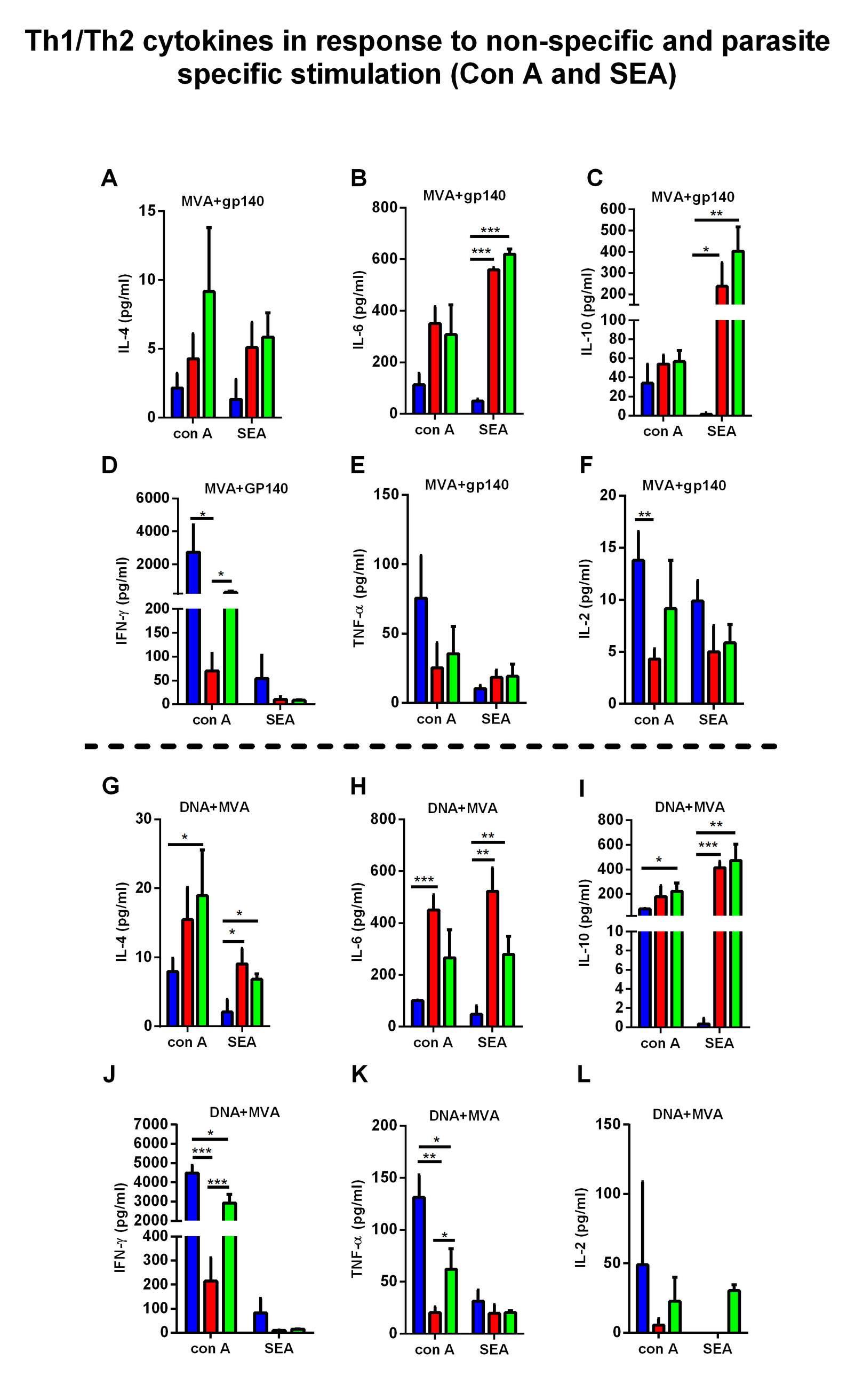

Supplement: S1 Fig — Splenocytes were harvested from mice vaccinated with the indicated regimen described in Fig 1. They were then stimulated with an irrelevant peptide (negative control), Con A or with SEA for 48 hours. Culture supernatants were collected and the level of Th1 and Th2 cytokines released into the medium for MVA+gp140 (A-C and D-F respectively) and DNA+MVA (G-I and J-L respectively) vaccinated mice was measured using a cytokine bead array assay. The individual bars represent the magnitude of the net cytokine levels for vaccinated Sm-free (blue); vaccinated Sm-infected (red) and Sm-infected-PZQ treated (green) vaccinated mice. Results represent 3 independent experiments and plotted as the mean + SEM, and cytokine levels were expressed as pg/ml. Statistical analysis was performed using unpaired, two-tailed t-test analysis followed by FDR for multiple comparisons. (*: p>0.05; **: p<0.01; ***: p<0.001). (TIF) [file ppat.1007182.s001.TIF]

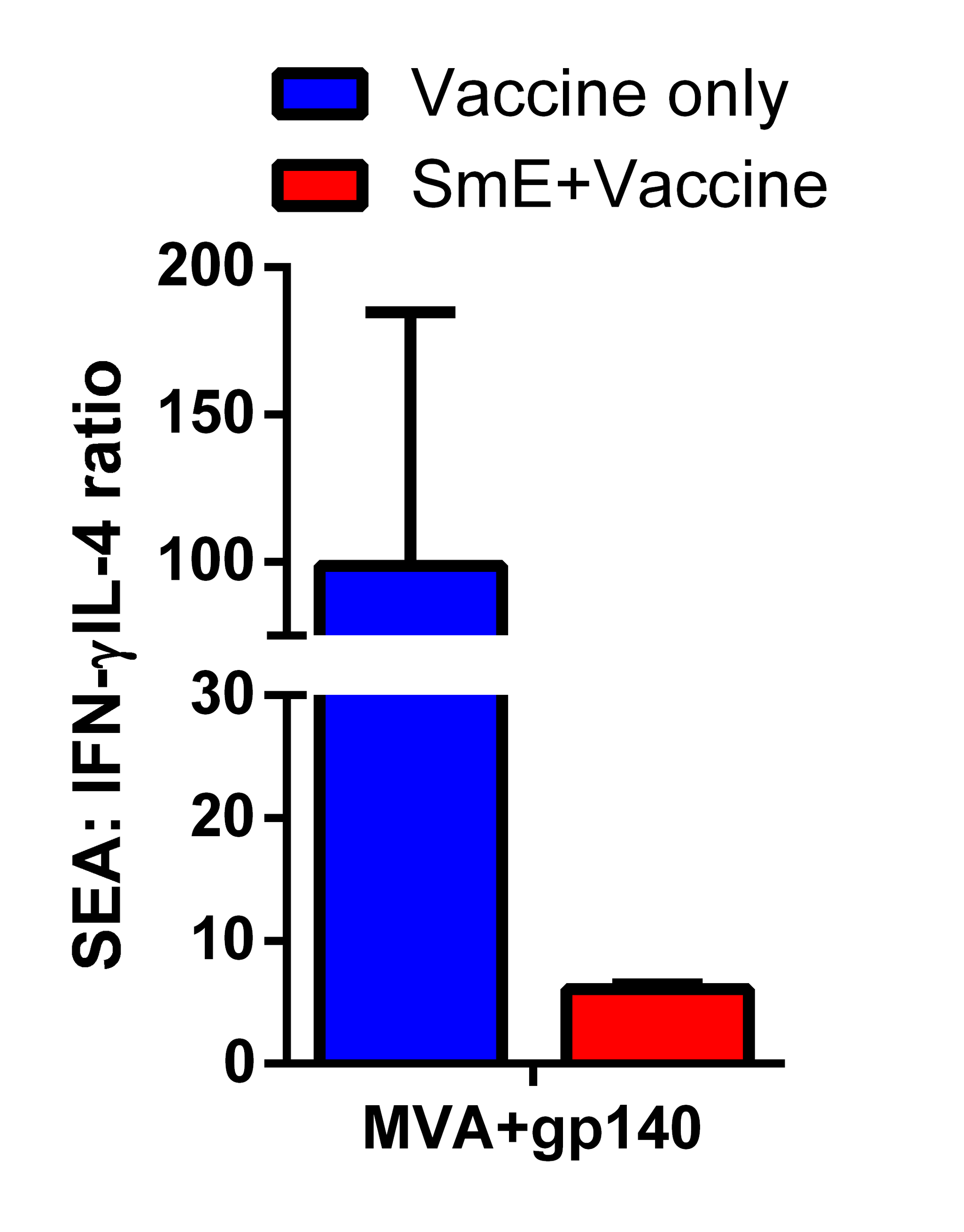

Supplement: S2 Fig — Splenocytes were obtained from SmE-sensitized and non-sensitized mice after two vaccinations with MVA-vectored HIV-1 and HIV-1 gp140 Env protein vaccines as shown in Table 2. They were then stimulated with an irrelevant peptide (negative control) or with SEA for 48 hours. Culture supernatants were collected and the level of Th1 and Th2 cytokines released into the medium was measured using a cytokine bead array assay. The individual bars represent the IFN-γ/IL-4 ratio for vaccinated non-sensitized (blue) and vaccinated SmE-sensitized (red) mice. Results represent 3 independent experiments and plotted as the mean + SEM. (TIF) [file ppat.1007182.s002.TIF]
