# Supplementary material for: Chronic schistosomiasis suppresses HIV-specific responses to DNA-MVA and MVA-gp140 Env vaccine regimens despite antihelminthic treatment and increases helminth-associated pathology in a mouse model
Source: PLoS Pathog. 2018 Jul 26;14(7):e1007182. doi: 10.1371/journal.ppat.1007182 (PMC6080792; doi:10.1371/journal.ppat.1007182)
Supplement: S1 Table — (DOCX) [file ppat.1007182.s003.docx]

S1 Table: Control and peptide stimulants used in the ELISpot, ICS and CBA assays

| **Stimulant** | **Source of stimulant** | **Description** | **Peptide sequence** |
| --- | --- | --- | --- |
| **Con A** | Sigma-Aldrich, USA | Non-specific polyclonal stimulus positive control | N/A |
| **Env CD4 (Env 6)** | Bachem, Switzerland | Env MHC class II-restricted CD4 epitope | -YGVPVWREAKTILFCA- |
| **Env CD8 (V3-CTL) peptide** | Bachem, Switzerland | Env H-2D^d^-restricted CD8 peptide | -RGPGRAFVTI- |
| **Gag CD4 (MRC13) peptide** | Bachem, Switzerland | Gag MHC class II-restricted peptide (CD4 peptide) | -NPPIPVGDIYKRWIILGLNK- |
| **Gag CD4 (MRC17) peptide** | Bachem, Switzerland | Gag MHC class II-restricted peptide (CD4 peptide) | -FRDYVDRFFKTLRAEQATQE- |
| **Gag CD8 peptide** | Bachem, Switzerland | Gag H-2K^d^ – restricted class I peptide (CD8 peptide) | -AMQMLKETI- |
| **Irrelevant peptide** | Bachem, Switzerland | Negative peptide control | -H-TXSTVASSL-OH- |
| **RT CD4 peptide** | Bachem, Switzerland | MHC class II-restricted CD4 peptide | -PKVKQWPLTEVKIKALTAI- |
| **RT CD8 peptide** | Bachem, Switzerland | H-2Kd-restricted RT peptide | -VYYDPSKDLIA - |
| **SEA** | Theodore Bilharz Research Institute, Egypt | Soluble Egg Antigen: (crude extract of heterogeneous proteins) | N/A |
